# Promising Low-Toxicity of Viologen-Phosphorus Dendrimers against Embryonic Mouse Hippocampal Cells

**DOI:** 10.3390/molecules181012222

**Published:** 2013-09-30

**Authors:** Joanna Lazniewska, Anna Janaszewska, Katarzyna Miłowska, Anne-Marie Caminade, Serge Mignani, Nadia Katir, Abdelkrim El Kadib, Maria Bryszewska, Jean-Pierre Majoral, Teresa Gabryelak, Barbara Klajnert-Maculewicz

**Affiliations:** 1 Department of General Biophysics, Faculty of Biology and Environmental Protection, University of Lodz, Pomorska 141/143, Lodz 90236, Poland; 2 Laboratoire de Chimie de Coordination CNRS, 205 Route de Narbonne, Toulouse 31077, France; 3 Laboratoire de Chimie et de Biochimie Pharmacologiques et Toxicologique, Université Paris Descartes, PRES Sorbonne Paris Cité, CNRS UMR 860, 45, rue des Saints Pères, Paris 75006, France; 4 Institute of Nanomaterials and Nanotechnology (INANOTECH)-MAScIR (Moroccan Foundation for Advanced Science, Innovation and Research), ENSET, Avenue de l’Armée Royale, Madinat El Irfane, Rabat 10100, Morocco

**Keywords:** apoptosis, cytotoxicity, hippocampal cell line, ROS, viologen-phosphorus dendrimers

## Abstract

A new class of viologen-phosphorus dendrimers (VPDs) has been recently shown to possess the ability to inhibit neurodegenerative processes *in vitro*. Nevertheless, in the Central Nervous Systems domain, there is little information on their impact on cell functions, especially on neuronal cells. In this work, we examined the influence of two VPD (VPD1 and VPD3) of zero generation (G0) on murine hippocampal cell line (named mHippoE-18). Extended analyses of cell responses to these nanomolecules comprised cytotoxicity test, reactive oxygen species (ROS) generation studies, mitochondrial membrane potential (ΔΨm) assay, cell death detection, cell morphology assessment, cell cycle studies, as well as measurements of catalase (CAT) activity and glutathione (GSH) level. The results indicate that VPD1 is more toxic than VPD3. However, these two tested dendrimers did not cause a strong cellular response, and induced a low level of apoptosis. Interestingly, VPD1 and VPD3 treatment led to a small decline in ROS level compared to untreated cells, which correlated with slightly increased catalase activity. This result indicates that the VPDs can indirectly lower the level of ROS in cells. Summarising, low-cytotoxicity on mHippoE-18 cells together with their ability to quench ROS, make the VPDs very promising nanodevices for future applications in the biomedical field as nanocarriers and/or drugs *per se*.

## 1. Introduction

The first reports on viologen-containing dendrimers were mainly focused on their electrochemical properties, resulting from the incorporation of electroactive viologen units into the dendritic structure [1,2,3]. The first report on biological properties of viologen-based dendrimers concerned their antiviral activities against a set of diverse viruses, and showed that they possess anti-HIV-1 activity [4,5]. Further interest in this class of dendrimers led Majoral’s team to the synthesis of original nanomolecules characterised by the presence of both viologen groups and phosphorus atoms in dendrimer branches, called viologen-phosphorus dendrimers (VPDs) [6]. Viologens (salts of 4,4'-bipyridine) are toxic compounds, causing strong generation of reactive oxygen species (ROS) in organisms. They were commonly used as very effective but non-specific herbicides [7]. However, these compounds were also responsible for severe human poisonings and seem to be responsible for development of Parkinson’s disease in case of prolonged exposure [8,9]. Cationic phosphorus dendrimers (CPDs) were demonstrated to possess very interesting biomedical applications being active *per se*, as transfecting agents [10], or as imaging agents for diagnosis [11]. Moreover, they were shown to exhibit anti-HIV properties [12] and potential to inhibit neurodegenerative processes *in vitro* [13,14]. Unfortunately, these promising nanomolecules are also characterised by relatively high cytotoxicity [15,16]. Combining viologen groups and phosphorus-containing dendrimers into one structure gave new qualities of the particles. An array of biological properties of these compounds were determined, including toxicity to mouse neuroblastoma (N2a) cells, Chinese hamster fibroblasts (B14), and erythrocytes, as well as antibacterial and antifungal activity [6]. The impact of VPDs on cellular processes in neuroblastoma cells was also investigated. These, compounds were shown to induce neither apoptosis nor necrosis, and cause only minor alterations in cell functions [17].

Recently, an increasing number of studies (*in vitro* and *in vivo*) have been focused on the potential of dendrimers to prevent aggregation and fibrillation of proteins involved in neurodegenerative disorders such as Alzheimer’s and Parkinson’s diseases and prion infections [13,18,19,20,21]. In addition, VPDs were examined for their ability to influence neurodegenerative processes *in vitro*. Indeed, they were shown to inhibit α-synuclein fibrillation, a process occurring in Parkinson’s disease [22], as well as to modify the activity of cholinoesterases associated with Alzheimer’s disease [23]. 

Importantly, some of the dendrimers were demonstrated to cross blood-brain barrier [24,25,26], which legitimised research on these compounds as potential drugs for neurological disorders. Recent *in vivo* studies have revealed that polyamidoamine (PAMAM) dendrimers possess the intrinsic ability to localise in cells associated with neuroinflammation (activated microglia and astrocytes) [27] and thus can be used as drug carriers in neuroinflammation therapy [28,29].

**Figure 1:**
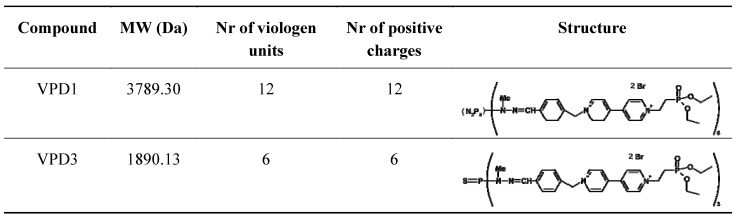
The structure and molar weight of tested viologen-phosphorus dendrimers.

Previously, we showed that treatment of cancerous N2a cell line with VPDs had only minor effect on the cells’ condition [17]. Nevertheless, there are no reports on their impact on normal neural cells. Therefore, as a continuation of our previous work, we decided to investigate the cell response to the treatment with VPDs on normal mouse hippocampal cell line (mHippoE-18). The choice of this cell line [30] was caused by the fact that hippocampus is a part of the brain affected by neurodegenerative diseases [31], and VPDs, as mentioned above, exhibit a potential to inhibit Alzheimer’s- and Parkinson’s-related processes. In our studies, we chose two water soluble VPDs (VPD1 and VPD3) of zero generation (G0). These nanomolecules possess the same type of surface groups (phosphonate moieties) but different core structures – hexa- or trifunctionalized in VPD1 and VPD3, respectively (Figure 1).

We investigated several cell processes after treatment with VPDs, which included reactive oxygen species (ROS) production, changes in oxidative activity of mitochondria, alterations in mitochondrial membrane potential (ΔΨm), induction of apoptosis/necrosis, morphological changes, cell cycle distributions, as well as the activity of catalase (CAT) and the level of reduced glutathione (GSH).

## 2. Results and Discussion

### 2.1. Cell Viability

**Figure 2:**
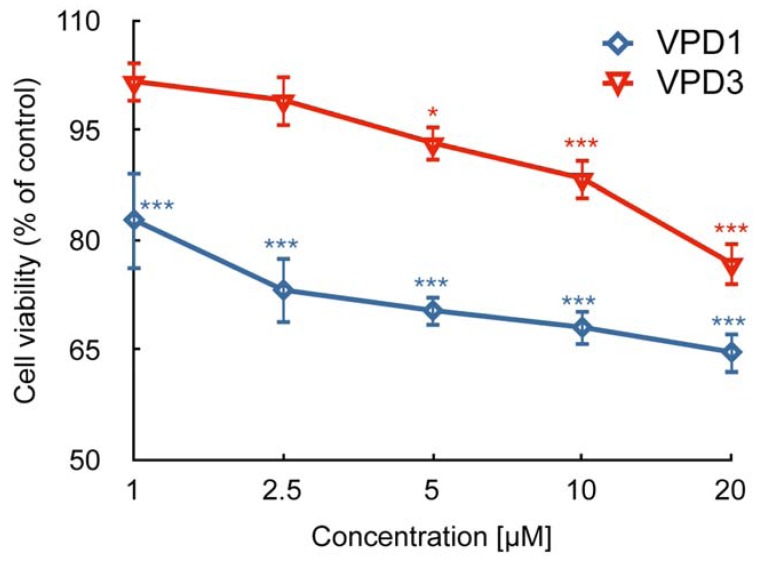
Cell viability of mHippoE-18 cells after 24 h treatment with VPDs (n = 5, * *p* < 0.05, ** *p* < 0.01, *** *p* < 0.001). Statistical differences occur for VPD1 between concentrations 1 and 2.5–20 μM, 2.5 and 20 μM, and for VPD3 between 1 and 5–20 μM, 2.5 and 10–20 μM, 5 and 20 μM, as well as 10 and 20 μM. Moreover, there are statistical differences between the two dendrimers at all tested concentrations.

It was demonstrated that VPD1 and VPD3 exhibited different toxicity against B14 and N2a cell lines. VPD1 decreased cell viability to about 50% of control in both cell lines. On the other hand, VPD3 reduced the viability of B14 to 80% of control, while its toxicity to the cancerous cell line was much stronger (decreased cell viability to 40% of control) [6]. We previously showed that both VPD1 and VPD3 reduced the viability of N2a cells to about 70% of control [17] and explained that discrepancies in our results and those obtained by Ciepluch *et al*. [6] may have resulted from different experimental conditions. In this work, we demonstrated that 24 h treatment of mHippoE-18 cells with VPDs led to the concentration-dependent loss of cell viability (Figure 2).

The effect was stronger in the case of VPD1, which possess more viologen units, positive charges and higher molar weight (see Figure 1). The highest concentration of VPD1 (20 μM) decreased cell viability to 64.64% of control, while 20 μM of VPD3 reduced the percentage of viable cells to 76.75% of control. In comparison to the results that we obtained for N2a cells, mHippoE-18 cell line appear to be slightly more sensitive to VPD1 but less to VPD3. Still, their cytotoxicity is very low in comparison to cationic dendrimers, such as PAMAM or CPDs [16,32]. The cytotoxicity of cationic dendrimers was proved to be dependent on the number of positive charges on the surface (thus, also generation of the dendrimer), since neutral and anionic counterparts were shown be much less cytotoxic [33,34,35]. Moreover, this charge (generation)-dependent cytotoxicity is postulated to result, at least partly, from the ability of dendrimers to cause perforation of the plasma membrane [36,37]. On the other hand, the interaction of dendrimers with the cell membrane is also a condition of their internalisation via endocytosis and trafficking to intracellular compartments [38]. It is likely that low toxicity of VPDs is related to the small number of positive charges and their location, *i.e*. not at the surface but inside the molecule.

### 2.2. ROS Formation and Dysfunction of Mitochondria

Excessive production of ROS is a common reaction of cells to stressful stimuli, and participates in cell-death associated signalling [39,40]. This cell reaction was also shown to occur during the treatment of neural cells with cationic dendrimers such as PAMAM and CPDs, and was associated with apoptotic (in mouse embryonic fibroblast cells) or necrotic (in mouse neural cells) cell death, respectively [16,41].

**Figure 3:**
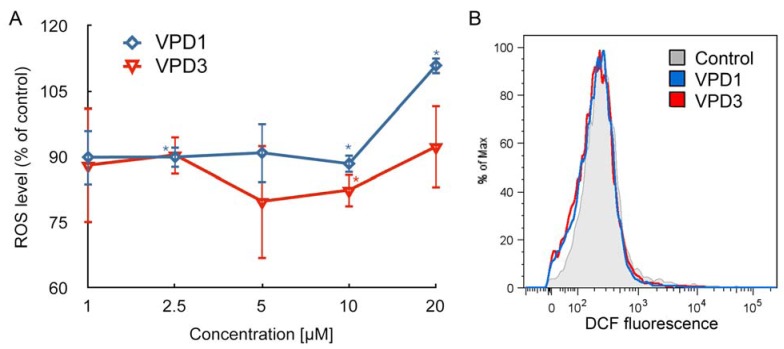
Alterations in the level of reactive oxygen species (ROS) after 24 h exposure of mHippoE-18 cells to VPDs (**A**) (n = 3, * *p* < 0.05). H_2_O_2_ was used as a positive control (133.02 ± 12.63 *). (**B**) is a representative histogram showing DCF fluorescence intensity in the control and samples treated with 10 μM of VPDs. There is a statistical difference for VPD1 between concentrations 10 and 20 μM.

Contrary to PAMAM and CPD dendrimers, VPDs were shown to slightly decrease the ROS level in N2a cells [17]. Similar results were obtained in this study for mHippoE-18 cell line. Figure 3 depicts that both VPDs, at the concentration of 10 μM, caused slight, but statistically significant, decrease in the level of ROS, which fell to 88.41% and 82.39% of control for VPD1 and VPD3, respectively. However, 20 μM of VPD1 led to a small statistically significant increase in DCF fluorescence, which reached 110.90% of control. This result indicates that higher concentrations of the dendrimer possessing more positive charges and viologen units can induce ROS generation. There are several examples in literature showing quenching of ROS in cells after dendrimer application. This process was observed in U-937 human macrophages treated with polypropyleneimine (PPI) dendrimers [42], as well as in Chinese hamster ovary (CHO) and human ovarian carcinoma (SKOV3) cells treated with PAMAM and PPI dendrimers [43]. Decrease or increase in ROS production compared to the control, depending on the dendrimer concentration and generation was also observed in both human keratinocytes (HaCaT) cell line and a primary adenocarcinoma cell line of colon (SW480) after treatment with PAMAM dendrimers [44,45]. Usually, ROS generation and mitochondrial membrane depolarisation or hyperpolarisation, beside other processes, accompany apoptosis [46,47]. In the above examples, despite the fact that ROS level was decreased, changes in ΔΨm occurred, which corresponded to the induction of apoptotic cell death, at least at some time points and doses.

**Figure 4:**
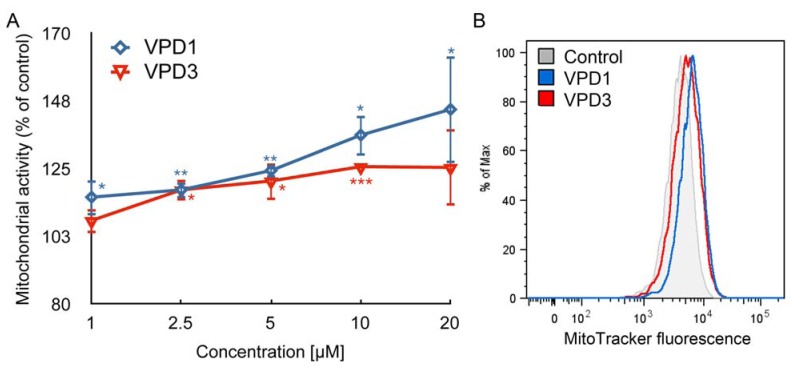
Alterations in oxidative activity of mitochondria in mHippoE-18 cells after 24 h treatment with VPDs (**A**) (n = 3, * *p* < 0.05, ** *p* < 0.01, *** *p* < 0.001). PPI was used as a positive control (231.59 ± 9.11 **). (**B**) is a representative histogram showing MitoTracker Orange fluorescence intensity in the control and samples treated with 20 μM of VPDs. Statistical differences occur for VPD1 between concentrations 1 and 20 μM, as well as 2.5 and 20 μM.

To obtain more detailed picture of VPDs mode of action, we also measured oxidative activity of mitochondria. Our results demonstrate that mitochondrial activity was increased along with the increasing concentration of VPDs (Figure 4). On the contrary, mitochondrial activity remained unchanged or was slightly decreased in N2a cells in response to VPDs [17].

**Figure 5:**
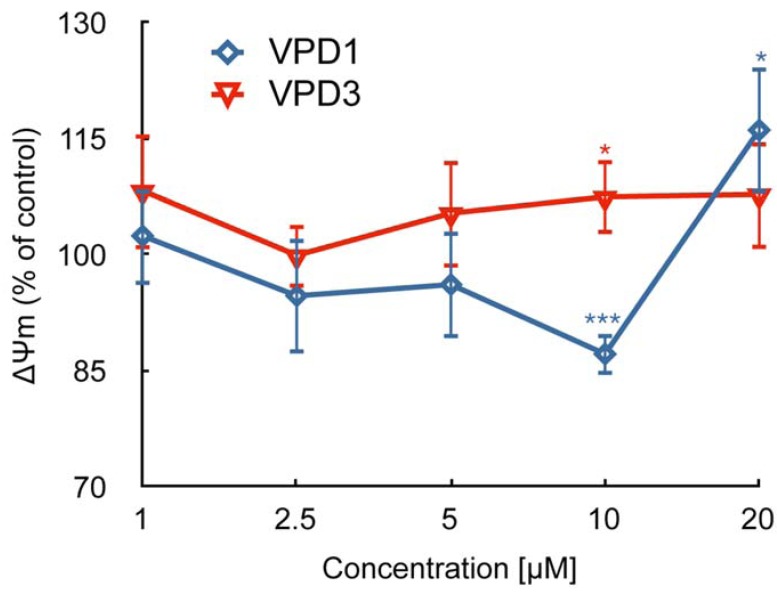
Mitochondrial membrane potential (ΔΨm) alterations in mHippoE-18 cell line after 24 exposure to VPDs measured using JC-1 probe (n = 5, * *p* < 0.05, ** *p* < 0.01,*** *p* < 0.001). CCCP was used as a positive control (46.21 ± 5.68 ***). There are statistical differences for VPD1 between concentrations 1 and 10 μM, 1 and 20 μM, as well as between VPD1 and VPD3 at the concentration of 10 μM.

Moreover, VPDs treatment for a mHipppoE-18 cell line caused perturbations in ΔΨm. In the case of VPD1 ΔΨm decreased to 87.13% of control at the concentration of 10 μM, while 20 μM caused small hyperpolarisation of the mitochondrial membrane (116.08% of control). VPD3 treatment led to the slight increase in ΔΨm, to 107.45% of control (Figure 5).

### 2.3. Cell Death Studies

**Figure 6:**
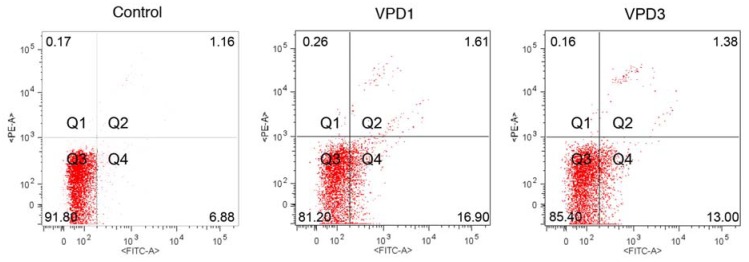
Representative dot plots showing fractions of healthy (Q3), apoptotic (Q4), and dead (Q1 and Q2) cells after 24 h exposure to 20 μM of VPDs.

Described above alterations in mitochondrial functions correspond to the small induction of apoptosis in hippocampal cells. Flow cytometric analysis of double-stained (YO-PRO1/PI) samples revealed that after VPD1 treatment, the fraction of viable cells decreased from 90.06% for the control to 85.43 and 81.70% at concentrations of 10 and 20 μM, respectively. Simultaneously, the population of apoptotic cells at these concentrations increased from 6.85% for control to 11.9 and 13.4%, respectively. VPD3 application to mHippoE-18 cells led to the fall in the percentage of viable cells at concentrations of 5 and 20 μM, and a slight increase in fraction of apoptotic cells at concentrations of 1, 2.5, 5 and 20 μM (Table 1, Figure 6).

**Table 1:** Detection of healthy, apoptotic and dead hippocampal cells after 24 h exposure to VPDs (n = 3, * *p* < 0.05, ** *p* < 0.01, *** *p* < 0.001). There are statistical differences for VPD1 between concentrations 1–5 and 20 μM for viable cells, 2.5 and 20 μM, as well as 5 and 20 μM for apoptotic cells.

	Control	VPD1				
C [μM]	0	1	2.5	5	10	20
**Healthy**	90.06 ± 1.53	87.70 ± 2.00	88.00 ± 1.06	89.37 ± 0.46	85.43 ± 0.31 **	81.70 ± 1.23 ***
**Apoptotic**	6.85 ± 0.86	9.34 ± 2.10	8.27 ± 2.92	7.44 ± 0.99	11.90 ± 1.01 ***	13.40 ± 2.40 **
**Dead**	2.51 ± 1.29	2.91 ± 0.81	3.75 ± 2.13	2.22 ± 0.57	2.77 ± 0.87	3.74 ± 1.66
	**Control**	**VPD3**				
**C [μM]**	**0**	**1**	**2.5**	**5**	**10**	**20**
**Healthy**	90.06 ± 1.53	87.07 ± 2.76	86.40 ± 2.87	86.57 ± 2.54 *	87.17 ± 2.02	86.50 ± 0.40 **
**Apoptotic**	6.85 ± 0.86	9.68 ± 0.92 **	8.96 ± 0.58 *	9.49 ± 1.43 *	8.55 ± 1.36	9.42 ± 0.15 **
**Dead**	2.51 ± 1.29	3.23 ± 1.90	5.40 ± 0.85 *	4.27 ± 0.64	4.39 ± 0.48	4.05 ± 0.33

**Figure 7:**
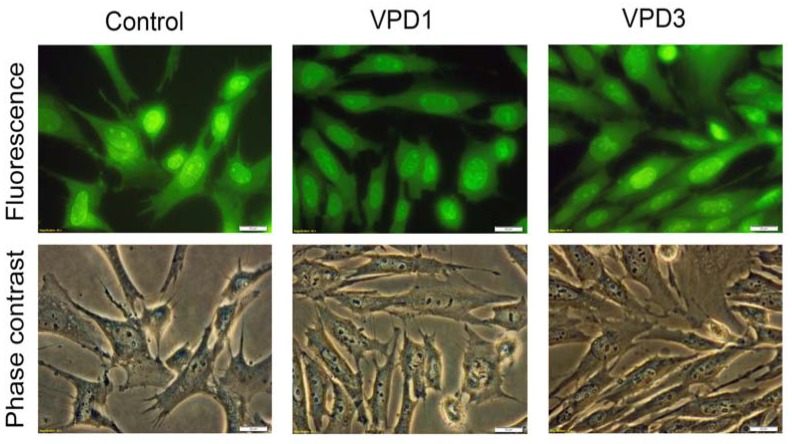
Fluorescence/phase contrast microscopy analyses of mHippoE-18 cells after 24 h exposure to 20 μM of VPDs. Magnification 400×.

Using fluorescent microscopy for the analysis of AO/EB-stained samples, it was difficult to distinguish VPD-treated cells from control ones (Figure 7, upper panel), since only single cells characterised by apoptotic morphology were observed in the field of view. This confirms that VPDs activated apoptosis only to a small degree. In contrast, N2a cells did not enter any cell death pathway in response to VPDs [17]. This suggests that in hippocampal cells formation of mitochondrial ROS, which did not occur in N2a cell line, together with changes in ΔΨm contributed to apoptosis induction. Obtained results are in agreement with previous reports, demonstrating that PAMAM-induced apoptosis was associated with the collapse of mitochondrial functions [48,49].

Moreover, both double-staining methods showed that the membrane integrity was maintained, since neither PI nor EB permeated into cells. This interesting result suggests that VPDs do not form nanoholes in the plasma membrane, and their internalisation rather occurs via endocytosis, as it was shown for other dendrimers [35,50,51].

### 2.4. Catalase (CAT) Activity and GSH Level Measurements

**Figure 8:**
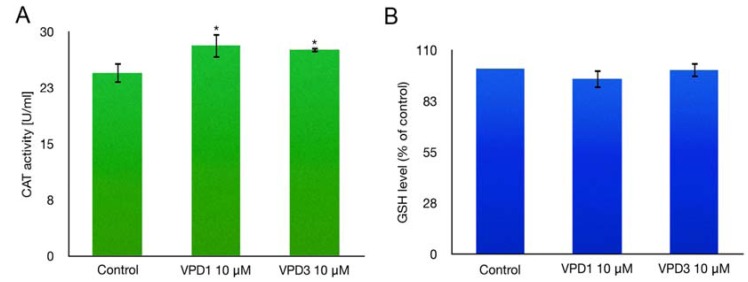
Catalase (CAT) activity (**A**) and level of reduced glutathione (GSH) (**B**) in mHippoE-18 cells after 24 h exposure to 10 μM of VPDs.

In a HaCaT cell line, changes of ROS generation were shown to be correlated with GSH depletion [45]. In order to check if the decline in ROS levels in mHippoE-18 cells after VPDs treatment corresponded to the activation of antioxidative systems of the cell, we monitored the level of GSH and CAT activity. Both VPDs at the concentration, which led to the fall in ROS level (10 μM), caused small, but statistically significant, increase in CAT activity (Figure 8A). CAT activity was elevated from 24.46 U/mL for the control to 28.15 and 27.62 U/mL for VPD1 and VPD3, respectively. CAT activity may increase in response to elevated ROS, which are then decreased by the enzyme to the value that is below the control level. Moreover, direct interactions between VPDs and CAT cannot be excluded. Indeed, it was shown that dendrimers, including VPDs, can influence the activity of various enzymes, such as cholinesterases, ATPase, or pepsin [23,52,53,54]. On the other hand, the level of the main cellular antioxidant - GSH, was not altered by testing VPDs in mHippoE-18 cell line (Figure 8B). Thus, it seems that mainly catalase is involved in ROS scavenging after VPDs treatment, although other antioxidative systems, which were not examined here, may also participate in this process.

### 2.5. Analyses of Cell Morphology

**Figure 9:**
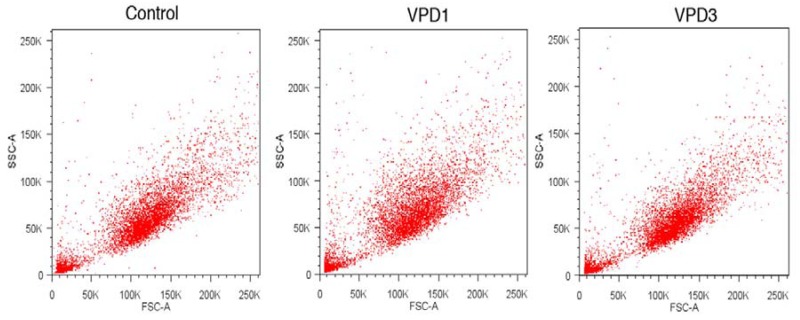
Representative dot plots showing size and granularity of mHippoE-18 cells after 24 h treatment with 20 μM of VPD.

Phase contrast microscopy analyses revealed that most cells maintained normal morphology after the treatment with 20 μM of VPDs (Figure 7, lower panel). Although cells characterised by apoptotic morphology were observed, their number was not markedly different from the control samples. Similarly, flow cytometric results did not reveal any important changes in cell size and granularity at the highest tested concentration of VPDs (Figure 9). This is understandable, since changes in the cellular processes caused by VPDs were not drastic and apparently did not influence cell morphology.

### 2.6. Cell Cycle Analysis

Since many cytotoxic compounds can cause cell cycle disturbances correlated with the cell death [55], we decided to check whether VPDs influence cell cycle profiles. Our results indicate that both VPDs caused small, although statistically insignificant, changes in cell cycle distributions (Table 2). Again, these results can be explained by only small level of apoptosis induced by VPDs, which is undetectable in cell cycle profiles.

**Table 2:** Analysis of cell cycle phases distribution in mHippoE-18 cell line after 24 h exposure to VPDs (n = 3, * *p* < 0.05, ** *p* < 0.01, *** *p* < 0.001).

	Control	VPD1				
[%]	0	1	2.5	5	10	20
**G0/G1**	51.38 ± 6.25	53.19 ± 5,75	52.76 ± 3.23	50.99 ± 6.24	45.62 ± 4.06	44.65 ± 5.22
**S**	26.33 ± 5.21	24.02 ± 4.3	26.32 ± 3.58	28.28 ± 4.26	31.88 ± 6.05	36.57 ± 6.34
**G2/M**	21.33 ± 1.42	22.9 ± 1.23	20.56 ± 2.27	22.27 ± 2.1	22.02 ± 2.20	18.62 ± 2.86
	**Control**	**VPD3**				
**[%]**	**0**	**1**	**2.5**	**5**	**10**	**20**
**G0/G1**	51.38 ± 6.25	54.07 ± 1.10	51.93 ± 2.82	50.33 ± 0.99	52.50 ± 2.02	53.87 ± 4.33
**S**	26.33 ± 5.21	22.83 ± 0.71	24.27 ± 1.10	24.67 ± 1.26	21.93 ± 0.38	21.13 ± 0.84
**G2/M**	21.33 ± 1.42	22.67 ± 1.18	23.00 ± 1.13	24.17 ± 2.14	24.57 ± 2.89	24.7 ± 3.49

## 3. Experimental

### 3.1. General

Viologen-phosphorus dendrimers were synthesized by the Laboratoire de Chimie de Coordination du CNRS. 3-[4,5-2-yl]-2-5-Diphenyltetrazolium bromide (MTT), 5,5′,6,6′-tetrachloro-1,1′,3,3′-tetraethylimidacarbocyanine iodide (JC-1), 2,7-dichlorodihydrofluorescin diacetate (H2DCFDA), acridine orange (OA), ethidium bromide (EB), camptothecin (CPT), propidium iodide (PI), pentachlorophenol (PCP), dimethyl sulfoxide (DMSO), hydrogen peroxide (H2O2), carbonyl cyanide 3-chlorophenylhydrazone (CCCP), RNase A, buffered saline (PBS) tablets, foetal bovine serum and trypsine were purchased from Sigma-Aldrich (St. Louis, MO, USA). MitoTracker Orange (CM-H2TMRos), YO-PRO-1 iodide, Amplex Red Catalase Assay Kit, ThiolTracker Violet dye, D-PBS C/M, and actinomycin D were purchased from Molecular Probes (Eugene, OR, USA). Dulbecco’s Modified Eagle Medium (DMEM) was purchased from Gibco (Eugene, OR, USA). 4th generation PPI dendrimers were obtained from Symo-Chem (Eindhoven, The Netherlands). All other reagents and solvents were of analytical grade.

### 3.2. Cell Culture

Embryonic mouse hippocampal cell line (mHippoE-18) was purchased from Cederlane (Burlington, Canada). Cells were cultured in DMEM medium supplemented with 10% foetal bovine serum and maintained at 37 °C in an atmosphere of 5% CO_2_. Cells were split for subcultures every 2–3 days. Cells were seeded in flat bottom 96-well plates either clear - for MTT assay, or black - for JC-1 and glutathione assays, at a density of 1.5 × 10^4^ cells/well in 100 μL of DMEM medium. For other assays cells were seeded in flat bottom12-well plates at a density of 2.5 × 10^5^ cells/well in 1 mL of DMEM medium. Cells were cultured during 20 h under growing conditions for cell attachment, and then treated with dendrimers.

### 3.3. Dendrimer Treatment

Dendrimer solutions were prepared using 10 mM PBS, pH 7.4. Cells were treated with five concentrations of a given dendrimer chosen based on previous works [6,17] (1, 2.5, 5, 10 and 20 μM). After 24 h dendrimer treatment, the supernatant was discarded, cells were washed with PBS (or D-PBD C/M in the case of ThiolTracker assay) and a given experiment was performed according to the procedures described below.

### 3.4. Cytotoxicity Assay

Cytotoxicity of viologen-phosphorus dendrimers was evaluated by MTT assay. The assay is based on the reduction of MTT by cellular reductases of viable cells to a blue formazan product, whose absorbance can be measured spectrophotometrically after solubilisation [56]. The assay was performed as previously described [57]. 0.5 mg/mL MTT was added to each well and incubated for 3 h in growing conditions. After this time MTT solution was discarded, DMSO was added to each well to dissolve formazan crystals and the absorbance was measured at 570 nm using a microplate spectrophotometer (BioTek, Burlington, VT, USA).

### 3.5. Measurement of Reactive Oxygen Species (ROS)

Changes in the level of reactive oxygen species (ROS) were measured using a fluorescent probe H_2_DCFDA. H_2_DCFDA does not fluoresce until it enters the cell, where the acetate groups are removed by intracellular esterases to form H_2_DCF. H_2_DCF is then oxidised to the fluorescent DCF [58]. Cells were stained with 500 μL of 2.5 μM H_2_DCFDA for 15 min in growing conditions. After staining the dye solution was removed, cells were washed with PBS, collected by trypsynisation and analysed by flow cytometry (LSRII, Becton Dickinson, Franklin Lakes, NJ) through the FL1 channel. Cells treated with H_2_O_2_ were used as a positive control. The data were recorded for a total of 10,000 events per sample.

### 3.6. Assessment of Oxidative Activity of Mitochondria

Mitochondrial activity was assessed using the reduced MitoTracker Orange (CM-H_2_TMRos) fluorescent probe. CM-H_2_TMRos is oxidised inside the cell to the fluorescent CMTMRos and then selectively sequestrated in mitochondria [59]. Cells were stained with 500 μL of 500 nM CM-H_2_TMRos for 30 min in growing conditions. After staining, the dye solution was removed, cells were washed with PBS, collected by trypsynisation and analysed by flow cytometry (LSRII, Becton Dickinson) through the FL2 channel. Cells treated with G4 polypropyleneimine dendrimers (PPI) were used as a positive control, which was established experimentally. The data were recorded for a total of 10,000 events per sample.

### 3.7. Assessment of Catalase (CAT) Activity

The catalase (CAT) activity was assessed using Amplex Red Catalase Assay Kit (Molecular Probes) according to the manufacturer’s protocol. 1 × 10^6^ cells were collected using a cell scraper. After centrifugation, cells were lysed for 30 min in 100 μL of cell extraction buffer on ice, centrifuged and the supernatant was collected. Cell lysate (50 fold diluted) was incubated with 40 μM H_2_O_2_ for 30 min. Then Amplex Red/HRP working solution was added to each well and incubated for 40 min. The fluorescence of the resorufin product was measured at λex = 530 nm, λem = 590 nm using a fluorescence microplate reader (Fluoroscan Ascent FL).

### 3.8. Assessment of Reduced Glutathione (GSH) Level

The cellular glutathione level (GSH) was estimated using ThiolTracker Violet dye, which reacts with reduced thiols in intact cells (Molecular Probes). Cells were incubated with 20 μM of ThiolTracker Violet dye for 30 min in growing conditions. Then cells were washed with D-PBS C/M and 100 μL of D-PBS C/M was added to each well. The fluorescence of the dye was measured at λex = 404 nm, λem = 526 nm using a Varian Cary Eclipse fluorimeter.

### 3.9. Assessment of Mitochondrial Membrane Potential (ΔΨm)

Mitochondrial membrane potential (ΔΨm) was determined using the fluorescent dye JC-1. JC-1 is a lipophilic cationic dye, which accumulates in mitochondria, where, at higher concentrations, it forms J-aggregates, which exhibit red fluorescence (λex = 530 nm, λem = 590 nm). When the mitochondrial membrane is depolarised, the dye does not form J-aggregates and exists in the form of monomers, which give green fluorescence (λex = 485 nm, λem = 538). The loss of ΔΨm can be indicated by a decrease in the red to green fluorescence intensity ratio [60]. 50 μL of 5 μM JC-1 was added to each well and incubated for 20 min in growing conditions. The dye was discarded, cells were washed with PBS and then 50 μL of PBS was added to each well. Fluorescence was measured using a fluorescence microplate reader (Fluoroscan Ascent FL). Carbonyl cyanide 3-chlorophenylhydrazone (CCCP) was used as a positive control.

#### 3.9.1. Determination of Apoptotic and Dead Cells by YO-PRO-1 Iodide/Propidium Iodide (PI) Staining-Flow Cytometry Analyses

YO-PRO-1 is a dye that can enter apoptotic cells, whereas PI cannot. Apoptotic cells show green fluorescence, dead cells show red and green fluorescence, and live cells show little or no fluorescence [61]. Cells were trypsinised, collected, YO-PRO-1 (0.1 μM) and PI (1.5 μM) were added to each sample and incubated for 20 min on ice. Then, the samples were analysed by flow cytometry (LSRII, Becton Dickinson) through the FL1 channel (530/30 bandpass filter) for YO-PRO-1 and FL2 channel (610/20 bandpass filter) for PI fluorescence. The data were recorded for a total of 10,000 events per sample. Compensation controls were prepared to induce apoptosis. Cells were treated with camptothecin (80 μM) for 4 h (positive green fluorescence), while to induce necrosis cells were treated for 1 h with pentachlorophenol (600 ppm) (positive red fluorescence). Actinomycin D was used as a double-stained positive control for apoptosis (data not shown).

#### 3.9.2. Visualisation of Cell Morphology, Apoptosis and Necrosis-Fluorescence/Phase Contrast Microscopy Analysis

OA/EB double staining was performed according to Ribble *et al.* [62] with some modifications. Acridine orange (AO) and ethidium bromide (EB) are DNA binding fluorescent dyes. The differential uptake of these dyes by cells allows for distinguishing viable cells from apoptotic and necrotic ones. AO enters all cells and stains the nucleus with green. EB permeates only damaged cell membranes and stains the nucleus with orange/red. Separate fractions of cells were identified as follows: viable cells—morphologically normal, green nucleus, early apoptotic cells—green nucleus with condensed or fragmented chromatin, late apoptotic cells—condensed or fragmented orange/red chromatin, necrotic cells—morphologically normal orange/red nucleus. Acridine orange (OA) and ethidium bromide (EB) were added to each well at a concentration of 2 μg/mL for 2 min. Cells were washed with PBS and visualised under fluorescence/phase contrast microscope (CKX41, Olympus, Tokyo, Japan) at magnification of 400×. The microscope was equipped with CCD camera (Olympus UC30). The used software was Olympus Stream version 1.6.

#### 3.9.3. Flow Cytometric Analysis of Cell Morphology

Cell morphology was also assessed using a flow cytometer (LSRII, Becton Dickinson). Cell size and granularity were determined with simultaneous separate detection of low-angle (FSC-A) and right-angle (SSC-A) light scattering. The data were recorded for a total of 10,000 events per sample.

#### 3.9.4. Cell Cycle Studies

Cell cycle distribution was analysed by flow cytometry (LSRII; Becton Dickinson) after PI staining according to Chang *et al*. [63] Cells were trypsinised; collected and fixed in ice-cold 96% ethanol for 24 h. Then; cells were washed with PBS and incubated for 30 min at 4 °C in 500 μL of staining solution containing 10 mM Tris-HCl (pH 7.5); 5 mM MgCl2; 10 μg/mL PI; and 10 μg/mL RNase A. After this time samples were analysed by flow cytometer (LSRII; Becton Dickinson) through the FL2 channel (610/20 bandpass filter). The data were recorded for a total of 10,000 events per sample.

#### 3.9.5. Statistics

For MTT, H2DCFDA, CM-H2TMRos, JC-1, and GSH assays the results were calculated relatively to untreated cells (100%) and differences between the control and the treated samples were evaluated using one-sample t-test, where *p* < 0.05 and below was accepted as statistically significant. For YO-PRO-1/PI, cell cycle, and CAT activity assays the differences between the control and the treated samples were evaluated using Student’s t-test, where *p* < 0.05 and below was accepted as statistically significant. For comparison between VPD1 and VPD3 and concentrations of the same VPD one-way ANOVA was performed followed by Tukey’s multiple comparison test. Data were presented as a mean ± SD of three to five individual experiments.

## 4. Conclusions

**Figure 10:**
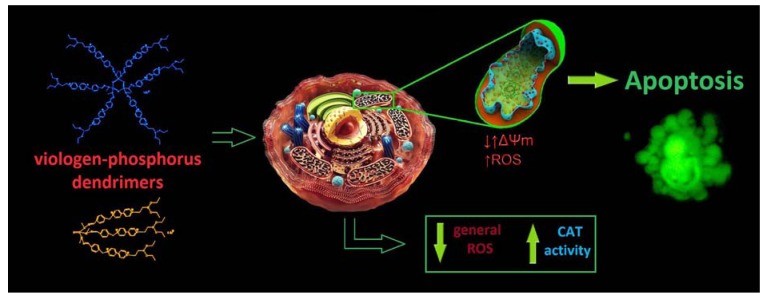
A schematic representation of the mechanism of action of VPDs on mHippoE-18 cells.

In light of recent findings that VPDs possess the ability to inhibit neurodegenerative processes *in vitro*, associated with, for instance, Alzheimer’s and Parkinson’s diseases [23,64,65] and the lack of data on the influence of these phosphorus dendrimers on neural cells, we aimed our research at studying the impact of two VPDs on the mouse hippocampal cell line. In this work, we demonstrated that tested VPDs are low-toxic to mouse mHippoE-18 cell line, and VPD3 was shown to be less harmful than VPD1. Both VPD1 and VPD3 did not cause serious changes in cell processes and led to only small increase in the apoptotic cell fraction. The small induction of apoptosis was shown to be correlated with mitochondrial dysfunction (Figure 10). Importantly, treatment of cells with these compounds resulted in the decreased ROS level, which corresponded to the increased CAT activity. This finding may be of high significance in the context of potential application of VPDs in biomedicine, for example, as drug carriers or active compounds *per se.* Overall, although more studies are needed, at this stage tested VPDs can be considered as relatively safe for mouse hippocampal cell line. Thus, they are promising macromolecules for further investigations on their applicability in biomedicine.
